# Cutaneous warmth, but not touch, increases muscle sympathetic nerve activity during a muscle fatigue hand-grip task

**DOI:** 10.1007/s00221-020-05779-x

**Published:** 2020-03-20

**Authors:** Rochelle Ackerley, Yrsa B. Sverrisdόttir, Frank Birklein, Mikael Elam, Håkan Olausson, Heidrun H. Krämer

**Affiliations:** 1 Clinical Neurophysiology, Sahlgrenska University Hospital, Göteborg, Sweden; 2 Department of Physiology, University of Gothenburg, Göteborg, Sweden; 3 Aix Marseille Univ, CNRS, LNSC (Laboratoire de Neurosciences Sensorielles et Cognitives-UMR 7260), Marseille, France; 4 Department of Neurology, University Medical Center, Johannes-Gutenberg University, Mainz, Germany; 5 Center for Social and Affective Neuroscience, Faculty of Health Sciences, Linköping University, Linköping, Sweden; 6 Department of Neurology, Justus-Liebig-University, Giessen, Germany

**Keywords:** Homeostasis, MSNA, Touch, Warmth, Vibration, C-fibre

## Abstract

In homeostasis, somatosensory C fibre afferents are hypothesised to mediate input to the brain about interactions with external stimuli and sympathetic efference provides the output that regulates bodily functions. We aimed to test this hypothesis and whether different types of innocuous somatosensory input have differential effects. Healthy volunteers performed a muscle fatigue (hand-grip) task to exhaustion, which produces increased muscle sympathetic nerve activity (MSNA), as measured through microneurography. Participants completed the muscle fatigue task without concurrent cutaneous sensory stimulation (control) or we applied skin warming (heat pack) as a C fibre stimulation, slow brush stroking as C and Aβ fibre stimulation, or vibration as Aβ fibre stimulation, to the participant’s forearm. We also measured heart rate, the duration of the hand-grip task, and ratings of pain at the end of the task. Concurrent skin warming showed increased MSNA compared to the other conditions. Tactile stimuli (brushing, vibration) were not significantly different to the control (no intervention) condition. Warming increased the pain from the muscle contraction, whereas the tactile stimuli did not. We interpret the effect of warming on MSNA as providing relevant afferent information during muscle contraction, which needed to be counteracted via vasoconstriction to maintain homeostasis. Brushing and vibration were less homeostatically relevant stimuli for the muscle contraction and hence had no significant effect. The findings add sensory specificity to our current understanding of homeostatic regulation through somatosensory afferent and sympathetic efferent pathways.

## Introduction

As we go about our daily activities, humans encounter external somatosensory stimuli that impact our body. In parallel, the efferent sympathetic system regulates various bodily processes to maintain homeostasis. It is hypothesised that C-fibre somatosensory afferents provide input to the insula about the state of the body and the sympathetic drive acts on this as required (Craig 2002, 2009). In general, muscle sympathetic nerve activity (MSNA) is associated with the control of blood flow and skin sympathetic nervous activity (SSNA) controls body temperature via sweat gland activity (Macefield 2013). It is well known that MSNA to skeletal muscle increases during muscle contraction and is related to the onset and duration of muscle fatigue (Seals and Victor 1991; Seals 1993). This has been studied under controlled conditions, where isometric muscle contraction (e.g. a hand-grip task) produces a clear increase in MSNA that can be recorded at a remote site (e.g. peroneal nerve of the leg), using multi-unit microneurography (Mark et al. 1985; Saito et al. 1986; Seals 1993).

MSNA is associated with the regulation of homeostatic mechanisms via vasoconstriction, especially in the cardiovascular system. During isometric exercise, MSNA is primarily increased via the muscle metaboreflex; however, MSNA may be altered by other factors. For example, MSNA can be modulated by respiration (Hagbarth and Vallbo 1968; Eckberg et al. 1985), changes in body position (Delius et al. 1972), and by mental stress (Wallin et al. 1973; Anderson et al. 1987). MSNA can also be altered by different stimuli applied to the body. The infusion of hypertonic saline causes long-lasting muscle pain and induces consistent increases or decreases in MSNA, depending on the individual (Kobuch et al. 2015), which may be due to a combination of incoming peripheral nociceptive signals and the way the individual reacts to this. MSNA has been shown to increase during painful heat and cold (Kregel et al. 1992; Lautenschläger et al. 2015). The onset of skin cooling can decrease the general level of MSNA (Kregel et al. 1992) and whole body warming has been shown to increase it (Niimi et al. 1997). Conversely, recording MSNA at sites local to the skin stimulation have found the opposite effect, where innocuous cold can increase MSNA (Ishida et al. 2016), whereas skin warming can decrease it (Takahashi et al. 2011), which was thought to be part of a local regulatory reflex. The activation of Aβ low-threshold mechanoreceptors via vibration can decrease MSNA (Strzalkowski et al. 2016), as can transcutaneous electrical skin stimulation (Goswami et al. 2012).

The above studies have measured the MSNA response induced from applying somatosensory stimuli directly to the skin, whereas less is known about how MSNA is regulated under ongoing stimulation. It has been demonstrated that in the presence of isometric exercise (hand-grip), local skin heating produces augmented MSNA (Ray and Gracey 1997; Kuipers et al. 2009). In contrast, the response to somatosensory stimuli different from local heating has been little investigated. As C-fibres are the afferent part of a homeostatic reflex arc while the autonomic nervous system is the efferent part (Craig 2002, 2009), we predicted that activating the C-fibre system to different degrees would modify MSNA. The present study focused on the body’s ability to react to and perceive applied somatosensory stimuli during ischemic muscle fatigue.

The present work utilises the technique of microneurography to record MSNA, while a hand-grip task was used to generate muscle exhaustion and concurrent somatosensory stimuli were applied to the arm. We aimed to preferentially stimulate C-warm fibres (and to some extent C-mechano-heat responsive nociceptors; Ackerley and Watkins 2018) through the application of a warm heat pack, C-tactile (CT) fibres through gentle stroking (Löken et al. 2009; Ackerley et al. 2014), and mechanoreceptive Aβ fibres using a vibratory stimulus (Strzalkowski et al. 2016). We hypothesised that the different somatosensory stimuli would modulate MSNA, physiological and behavioural measures differentially, where conditions with increased thin fibre afference would have stronger effects on the measures, due to their homeostatic relevance.

## Methods

The investigation was performed on healthy participants, in accordance with the World Medical Association Declaration of Helsinki, and following approval of the local ethics committee of the University of Gothenburg. Prior to the experiment, participants were given standard information and written informed consent was obtained. We used the in vivo technique of microneurography (Vallbo et al. 2004) to gain axonal recordings of vasoconstrictor outflow from humans during somatosensory stimulation with concurrent muscular fatigue, and additional physiological and behavioural measures were gained.

Multi-unit, MNSA microneurographical recordings were made from the left leg in 35 participants (age range 20–40 years; 20 male). MSNA has local muscle and body-wide influence, but in general, MSNA is similar across the body (Hansen et al. 1994; Ray and Mark 1995; Boulton et al. 2019). It is, therefore, possible to measure MSNA on a well-accessible site (e.g. the peroneal nerve of the leg), while the stimulation is carried out at another part of the body (e.g. the arm), as in the present study. Such a setup has been used in previous work (Saito et al. 1986; Seals and Victor 1991; Seals 1993; Ray and Mark 1995). A muscle fatigue hand-grip task was chosen as it is known to produce clear muscle exhaustion, but without exercise effects (e.g. increasing body temperature, sweating) and is effective in provoking MSNA.

The hand-grip dynamometer consisted of two wooden blocks joined together (size 10 × 5 cm) with a force monitor in the middle, which was capable of recording the time and pressure of the grip. Each participant first did a hand-grip muscle fatigue calibration. The calibration provided a standard level of muscle fatigue per participant, before commencing the experiment. Here, participants were instructed to apply their maximum hand grip for 60 s, during which a custom-written MATLAB (The Mathworks, Natick, MA, USA) program determined the 30% maximum voluntary contraction. This 30% maximum voluntary contraction represented the participant-specific grip level that the participant was required to maintain during the muscle fatigue experimental task until muscular exhaustion. Throughout the muscle fatigue task, hand-grip force was presented on a monitor to the participant, as well as the experimenter, so that it could be corrected continuously.

The hand-grip task was used as a muscle fatigue stimulus during four experimental conditions. Each participant completed two out of the four conditions, in one experimental visit. Thus, they completed one condition using the muscle fatigue task on one arm, then after a break, they completed a second condition, using the muscle fatigue task on the other arm. We chose not to conduct all four conditions in single participants, as microneurography combined with the fatigue task was already challenging, as the processes was mentally and physically tiring. Further, we did not want to perform the hand-grip muscle fatigue task on recently fatigued muscle and it is not recommended to perform microneurography in the same nerve without a gap of ~ 3 months.

The four conditions during a muscle fatigue task were (1) ‘control’, where the muscle fatigue task was completed alone with no concurrent intervention; (2) ‘warmth’, where a warming heat pack (size: 10 × 7 cm, average temperature 40 °C measured with a contact thermometer; Fluke; Germany) was placed on the arm doing the hand-grip; (3) ‘brush’, where slow brushing with a soft brush (back-and-forth brushing traversing 10 cm skin in ~ 1 s, brush width: 7 cm) was carried out on the arm doing the hand-grip; (4) ‘vibration’, where 50 Hz tactile vibration (vibrometer area: 4 × 1 cm) was carried out on the arm doing the hand-grip. Each one of these concurrent stimuli was carried out in the middle of the dorsal forearm and the concurrent somatosensory stimuli were applied on the same arm as the hand-grip muscle fatigue task. Participants were assigned to two out of the four conditions in a pseudo-random order. The first hand used for the muscle fatigue hand-grip task was randomised for order between (right or left) participants. The different somatosensory stimuli started at the beginning of the hand-grip and were applied during the entire duration of the hand-grip muscle fatigue task. Three types of responses were recorded in these experiments: (1) microneurography, multi-unit MSNA responses, (2) physiological haemodynamic responses, and (3) behavioural responses, as detailed below.

1. The multi-unit recordings were made with low-impedance, insulated tungsten electrodes (200 µm shaft and 5 µm tip diameters; FHC, Bowdoin, ME) inserted into the peroneal nerve, posterior to the fibular head. An uninsulated reference electrode was inserted sub-cutaneously nearby. For further details of the microneurography and MSNA criteria, see Vallbo et al. (1979, 2004) and Low (1997). Once the electrode had entered a muscle nerve fascicle (as indicted by responses to manual muscle stimulation and no response to skin touch), small adjustments were made in the electrode location to maximize recordings of spontaneous MSNA. During the experiment, MSNA data were amplified (50,000×), filtered (band pass 700–2000 Hz) and fed through a discriminator for audio monitoring. A mean voltage (integrated), smoothed signal was obtained by passing the nerve signal through a resistance–capacitance circuit (time constant 0.1 s). Burst identification was performed automatically and checked visually by the experimenter. Three MSNA measures were taken from this signal: the burst frequency (number of bursts per minute), the burst incidence (number of bursts occurring per hundred heartbeats) and the burst area (total neural activity as a product of the area under the burst over a minute, in arbitrary units). Burst incidence illustrates central control (the relative amount of cardiac cycles filled with MSNA bursts) whereas burst frequency reflects the degree of activity reaching the effector organs (Sundlöf and Wallin 1977).
2. Heart rate (measured as beats per minute) was taken before and during the task, using electrocardiography via chest electrodes. Mean arterial blood pressure was also measured (middle finger cuff; Finapres 2300, Louisville, KY, USA); however, this measure was only reliable in around half of the participants over the time course of the muscle fatigue task and was, therefore, only used to ensure normal haemodynamic function from readings taken at the beginning of the task, and not analysed further.
3. Two behavioural measures were recorded: the duration of the hand-grip (in seconds) of the muscle fatigue task, and a pain rating when the task ended. Participants rated their level of pain at the point of muscle exhaustion to the hand-grip on a visual analogue scale, with anchors from ‘no pain’ (equating to ‘0’ in our data) on the left to ‘most intense pain imaginable’ (equating to ‘100’) on the right.

MSNA and heart rate were recorded throughout the experiment and average baseline resting values for these were taken over a 5-min period before the first muscle fatigue task for each participant. After the first task was completed, the participant rested for at least 10 min and second average baseline values were gained over a 1-min period before starting the second muscle fatigue task on the other arm. The responses gained in a participant’s specific task were compared to the baseline period occurring directly before that task. In the analysis, the average MSNA for burst frequency, incidence, and area, as well as the heart rate, from both the first and last minute of the muscle fatigue task were compared to the specific task average baseline readings per participant. Due to the typical variation seen in these physiological responses, these data were normalised to the averaged baseline values (which were set at 100%). The MSNA and heart rate data presented in the current study show the average responses from the included participants as an increase or decrease from the 100% baseline measures.

### Data analysis

To compare differences and correlations in the data, statistical tests were carried out using SPSS (version 23; IBM, Armonk, NY, USA). The first analysis step was to check that the two pre-test baselines did not differ using paired *t* tests. As a stable baseline was required over time, the criterion for exclusion was if a participant had two or more measures that varied by > 30% in MSNA (out of the burst frequency, burst incidence, and burst area) or that the heart rate varied by > 10%, between both baselines. The majority of participants did not differ significantly; however, seven participants were excluded as they displayed inconsistent baseline physiological (MSNA and/or heart rate) measurements. Since ischemic muscle pain was seen as a prerequisite to move from homeostasis to provoke an autonomic response, participants with insufficient pain rating (< 5 on our numeric rating scale) were excluded as well. Two participants had insufficient pain ratings, but they were in the group of seven excluded participants, as they also showed inconsistent baseline physiological measurements. For the included participants, each of the MSNA, heart rate, and behavioural measures, per condition, was tested for normality using a Kolmogorov–Smirnov test and were found to be normally distributed. Statistical effects are presented at the *p* < 0.05 level and significance stated to three decimal places.

The second analysis step was to investigate whether MSNA and heart rate showed changes due to the muscle fatigue task. To assess changes over time, the baseline normalised MSNA and heart rate were compared to the same measure from (i) the first-minute of the task and (ii) the last-minute of the task, using one-sample *t* tests. Then the measures were entered into separate general linear model analyses of variance (ANOVAs) as dependent variables, with the condition type as the independent variable (with four levels corresponding to the different conditions: control, warm, brush, vibration). A full-factorial model was used to assess the main effect of condition on each of the measures, and the sex of the participant was included as a covariate. We also provide the effect sizes as partial Eta squared and the observed power (computed using alpha = 0.05). When a significant main effect was found, pairwise comparisons between the different conditions were tested using the Tukey method, which corrects the *p* value for the multiple comparisons. A correlation analysis was also conducted on the end-point responses from all the measures to determine which measures were positively or negatively correlated, using Pearson’s test.

## Results

**Fig. 1:**
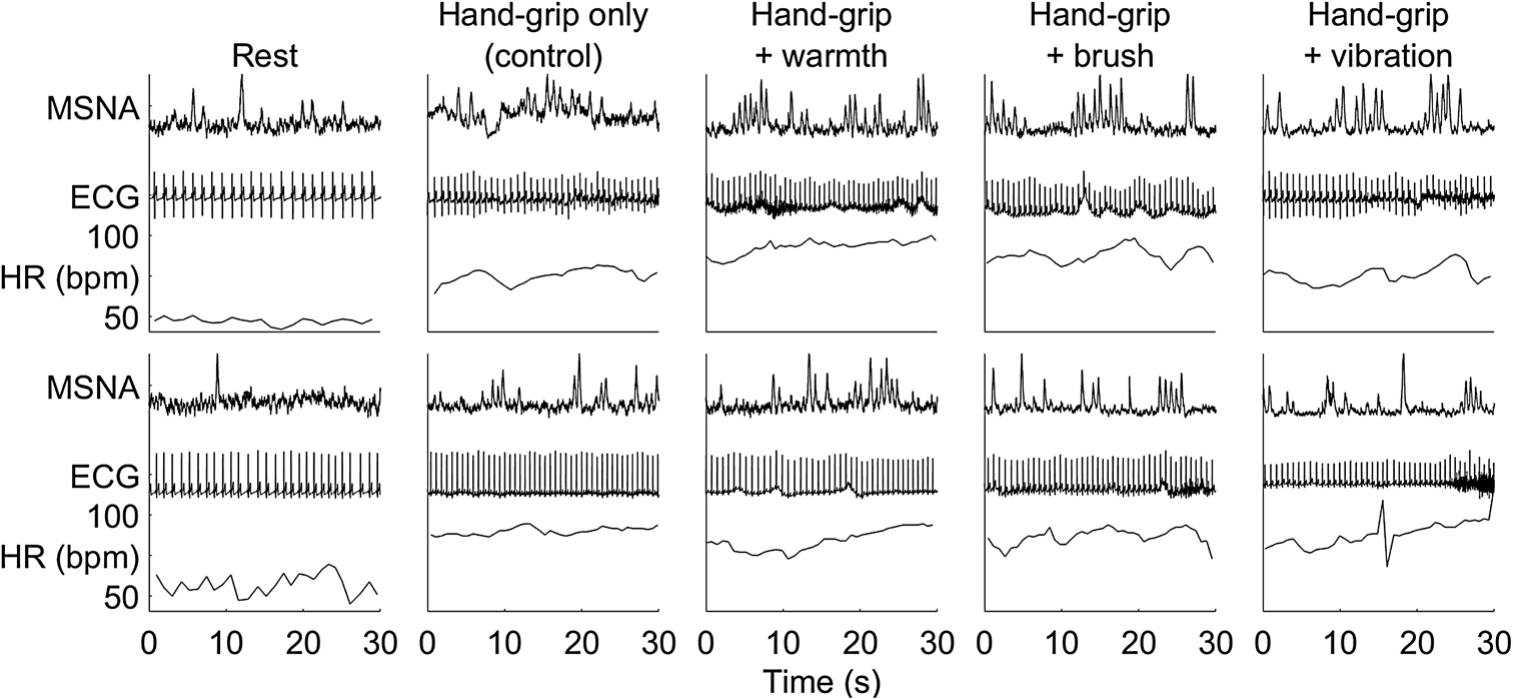
Examples of integrated MSNA, ECG, and heart rate. 30 s of continuous recordings from near the end of the task for integrated muscle sympathetic nerve activity (MSNA), electrocardiography (ECG), and heart rate (HR) in beats per minute (bpm) from four experiments, during (from left-to-right) rest (baseline), hand-grip only, and hand-grip with concurrent warmth, brushing, or vibration applied to the forearm. The data are from participant CB971121 (top row; rest, control, vibration), participant F07030 (top row; warmth, brush), participant ES071105 (bottom row; rest, brush, vibration) and participant AN100423 (bottom row; control, warmth)

The study was a cross-over design and 28 of the participants could be included in the full analysis. The numbers of participants in each condition were control (no intervention): *n* = 16, warm: *n* = 11, brush: *n* = 17 and vibration: *n* = 12. Examples of MSNA, electrocardiography, and heart rate from different participants are displayed in Fig. 1.

**Fig. 2:**
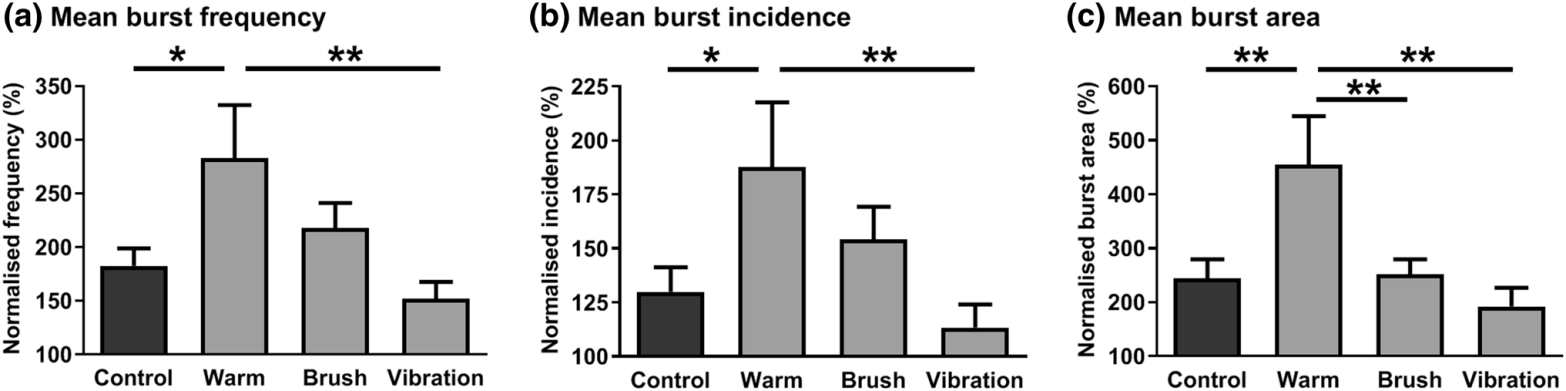
End-point MSNA responses to evoked muscle pain over the conditions. Microneurographic recordings of muscle sympathetic activity showing the normalised mean sympathetic **a** burst frequency, **b** burst incidence and **c** burst area. The responses were taken in the last minute of the hand-grip task for each participant and normalised to each participant’s pre-task resting baselines (100%). There was a significant increase in all of the MSNA measures over the conditions, as compared to the resting baseline (all *p* < 0.001). The significances shown indicate the between-condition effects from the separate measures (*p < 0.05, **p < 0.01). Error bars are +SEM

Overall, there was a significant increase in all the MSNA measures, compared to the resting baseline, for the hand-grip task and concurrent warmth, brushing, vibration and during no concurrent stimulation (all *p* < 0.001; Fig. 2). The MSNA recordings showed significant main effects for condition type in mean burst frequency (Fig. 2a, Table 1), mean burst incidence (Fig. 2b, Table 1), and mean burst area (Fig. 2c, Table 1). There were no effects of sex on any of the MSNA variables (Table 1). The main effect of condition relied mainly on the warm condition because this induced higher MSNA responses than the control and vibration condition for burst frequency (control: *p* = 0.013; vibration *p* = 0.003; Fig. 2a) and burst incidence (control: *p* = 0.023; vibration *p* = 0.007; Fig. 2b). Additionally, the warm condition induced a larger burst area than the brush condition (control: *p* = 0.003; vibration *p* = 0.001, warmth *p* = 0.004; Fig. 2c). No differences were found between the vibration, brush and control conditions.

**Table 1:** Statistical effects for condition and sex on each measure

Variable	Main effect of condition	Effect of sex as covariate
Mean burst frequency	***F***_**(3, 51)**_** = 3.66, p = 0.018** Effect size = 0.18, power = 0.77	*F*_(1, 51)_ = 0.06, *p* = 0.803 Effect size < 0.01, power = 0.06
Mean burst incidence	***F***_**(3, 51)**_** = 3.05, p = 0.037** Effect size = 0.15, power = 0.68	*F*_(1, 51)_ = 0.03, *p* = 0.856 Effect size < 0.01, power = 0.05
Mean burst area	***F***_**(3, 51)**_** = 5.12, p = 0.004** Effect size = 0.23, power = 0.90	*F*_(1, 51)_ = 0.07, *p* = 0.791 Effect size < 0.01, power = 0.06
Heart rate	*F*_(3, 51)_ = 0.79, *p* = 0.508 Effect size = 0.05, power = 0.21	*F*_(1, 51)_ = 3.30, *p* = 0.076 Effect size = 0.07, power = 0.43
Duration of hand grip	*F*_(3, 51)_ = 0.71, *p* = 0.550 Effect size < 0.01, power = 0.19	*F*_(1, 51)_ < 0.01, *p* = 0.997 Effect size < 0.01, power = 0.05
Pain intensity rating	***F***_**(3, 51)**_** = 3.05, p = 0.037** Effect size = 0.15, power = 0.68	*F*_(1, 51)_ = 0.62, *p* = 0.435 Effect size = 0.01, power = 0.12

The table shows the statistical details per test and the only significant effects found were for condition on all three MNSA variables and on pain intensity ratings (highlighted in bold)

**Fig. 3:**
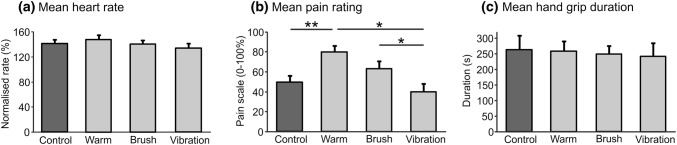
Heart rate, duration of hand-grip and end-point pain ratings over the conditions. **a** Normalised mean heart rate was taken in the last minute of the hand-grip task for each participant and normalised to each participant’s pre-task resting baselines (100%). There was a significant increase in heart rate over all of the conditions, as compared to the resting baseline (all *p* < 0.001), but not between-condition effects. **b** Mean pain ratings on a visual analogue scale ranging from 0 to 100 obtained at the end of each condition. There was a significant effect of condition on the pain ratings and between-condition significances are shown (*p < 0.05, **p < 0.01). Error bars are +SEM. **c** Mean hand-grip duration showed no significant effect of condition on the task duration (participants were instructed to continue to grip until exhaustion)

Heart rate increased during the last minute in all the conditions, as compared to the resting baseline (all *p* < 0.001; Fig. 3a). However, there were no between-condition effects for heart rate, or for sex (Table 1). The duration of the task did not differ between the conditions (Fig. 3c). Conversely, there was a significant main effect of the condition type on the end pain ratings (Fig. 3b, Table 1). In the warm condition, pain intensity was significantly higher than in the control and vibration conditions; additionally, the concurrent brushing condition showed increased pain ratings over the vibration condition (all *p* < 0.05; Fig. 3b). Again, there was no significant effect of sex on either the duration of hand grip or on the pain intensity rating.

A correlation analysis was conducted between all end-point measures consisting of MSNA, heart rate, and behavioural parameters. Table 2 shows the significant correlations between these measures in bold text. Many significant correlations were found: as expected, all of the MSNA measures were significantly positively correlated with each other, and the burst frequency and burst area correlated better with the other measures than the burst incidence. Heart rate correlated positively with all of the measures, except for the burst incidence. The pain ratings correlated significantly with all of the measures except for the task duration, and the task duration measure only correlated significantly with heart rate (for details, see Table 2).

**Table 2:** Correlations between the end-point physiological and behavioural measures

Measure	Burst frequency	Burst incidence	Burst area	Heart rate	Pain rating	Task duration
Burst frequency		**0.948** ***p < 0.001***	**0.855** ***p < 0.001***	**0.450** ***p = 0.001***	**0.408** ***p = 0.002***	0.094 n.s
Burst incidence			**0.800** ***p < 0.001***	0.159 n.s	**0.330** ***p = 0.013***	0.003 n.s
Burst area				**0.407** ***p = 0.002***	**0.324** ***p = 0.015***	0.095 n.s
Heart rate					**0.367** ***p = 0.005***	**0.290** ***p = 0.030***
Pain rating						0.049 n.s

Pearson’s *R* and significance values for each measure comparison are shown. Significant correlations are highlighted in bold

## Discussion

Concurrent somatosensory stimulation during a muscle fatigue hand-grip paradigm induced between-condition differences in MSNA, heart rate, and pain, with warmth inducing higher MSNA burst frequency, burst incidence, and burst area compared to the control and vibration condition, and higher burst area compared to the brush condition. MSNA parameters correlated with the experimentally induced pain intensity from muscle exhaustion, linking the central processing of the task with the MSNA regulatory output.

The concurrent sensory stimuli were chosen to preferentially activate different types of skin afferents as we hypothesised that increased thin-fibre afferents would have a larger homeostatic effect. The application of warmth to the skin is an effective C-fibre stimulus and this increased MSNA the most. However, we cannot say whether this mechanism was due to (1) the direct influence of increasing muscle temperature, (2) a more general reflex arc from somatosensory input provoking increased sympathetic output, or (3) a combination of these two and/or other mechanisms. Exercise increases local muscle temperature, which is transmitted by thin muscle afferents (Hertel et al. 1976; Kumazawa and Mizumura 1977) and this increases MSNA, but additional warmth augments it even further (Ray and Gracey 1997; Kuipers et al. 2009). Nearby skin heating may sensitise the muscle mechanoreflex induced from the hand grip and Kuipers et al. (2009) proposed that this mechanism caused increased MSNA during concurrent arm warming. Ray and Gracey (1997) postulated that the mechanism was through increased activity of mechanoreceptive muscle afferents that were also sensitive to temperature. However, these studies and our present work all find increased MSNA during warming with hand grip and it cannot be discounted that skin afferents, especially C-warm and C-mechano-heat-sensitive nociceptors (that can be activated through innocuous heat; Ackerley and Watkins 2018), will send innocuous afferent information about skin conditions. Therefore, if the participant feels warm, the corresponding somatosensory signal will be present.

Although we cannot be sure about the underlying mechanism behind warmth eliciting additional MSNA during hand grip, we provide new insights into the regulation of MSNA during muscle fatigue with other types of somatosensory stimuli. It is known that different somatosensory stimuli have specific afferent pathways, where touch and nociception are largely separate (McGlone et al. 2014; Marshall et al. 2019), although it is thought that the insula is a target for C-fibre afferents providing input about physiological body conditions (Craig 2002, 2003). Presently, we found no additional increases or decreases of MSNA with tactile stimulation during hand grip. A previous study showed decreased MSNA with vibration alone (Strzalkowski et al. 2016), but it seems that in our experiment, the overriding effect was an increase in MSNA due to the hand grip. However, MSNA was generally lower during vibration and was significantly lower than MSNA with concurrent warmth. Vibration, which effectively activates Aβ mechanoreceptive afferents, reduced pain in the hand-grip task, as compared to warmth or brushing, where decreased pain may lead to reduced MSNA. Transcutaneous electrical nerve stimulation activates large diameter cutaneous nerve fibres pain and can decrease pain, by modulating the central transmission of pain (Melzack and Wall 1965), especially for inputs converging on the same spinal level (Hollman and Morgan 1997). Such electrical skin stimulation can also decrease MSNA (Goswami et al. 2012) and thus may be more effective than vibration, but further work would need to be carried out to evaluate this.

We believed that brushing touch, which stimulates both C-fibre (CT afferents) and Aβ mechanoreceptive afferents (although is an effective CT stimulus, Löken et al. 2009; Ackerley et al. 2014), could have either increased MSNA (due to C-fibre activation and its involvement in homeostasis) or decreased it (due to such stroking being pleasant and relaxing). However, we found no significant effect during hand grip and that may have been due to competing effects. In contrast to a previous investigation on heat pain (Liljencrantz et al. 2017), the addition of CT-optimal stimulation by brushing was not sufficient to reduce pain from muscle fatigue in our hand-grip task. However, we found that MSNA burst area was significantly lower during brushing than warmth. Recent studies indicate that pathophysiological mechanisms mediating the occurrence and strength (burst area) of sympathetic bursts are different (Kienbaum et al. 2001). It has also been shown that whole body heating leads to an increase of MSNA activity, but leaves the burst area unchanged (Keller et al. 2006). Therefore, the decrease in MSNA burst area during brushing may be part of a wider homeostatic mechanism.

We conclude that warmth is an additional relevant homeostatic input because thermal signals are important during exercise, and need to be counteracted. The tactile stimuli did not decrease MSNA, as compared to the control, possibly because it did not provide a strong enough or relevant homeostatic drive that needed counteracting. Further studies could investigate whether SSNA changes under such conditions, as well as testing other somatosensory stimuli (e.g. cooling) and measuring local and non-local MSNA with and without isometric exercise, to see whether sympathetic efference can be modulated, which could help in situations such as physiotherapy and rehabilitation.

## Data Availability

Data are available by request from the authors.
